# Research trends in glioma chemoradiotherapy resistance: a bibliometric analysis (2003–2023)

**DOI:** 10.3389/fonc.2025.1539937

**Published:** 2025-02-07

**Authors:** Shishi Yu, Jinya Wu, Yuan Jing, Ping Lin, Lang Lang, Yifan Xiong, Wangzhong Chen, Wenhua Liu, Changpeng Sun, Yuntao Lu

**Affiliations:** ^1^ The Editorial Department of the Journal of Southern Medical University, Southern Medical University, Guangzhou, Guangdong, China; ^2^ Clinical Research Center, Tongji Hospital, Tongji Medical College, Huazhong University of Science and Technology, Wuhan, China; ^3^ Nanfang hospital, Southern Medical University, Guangzhou, Guangdong, China

**Keywords:** glioma, chemoradiotherapy resistance, tumor microenvironment, bibliometric analysis, immune infiltration, androgen receptor

## Abstract

**Background:**

Glioma is the most aggressive primary malignant tumor of the central nervous system, characterized by high recurrence rates and resistance to chemoradiotherapy, making therapeutic resistance a major challenge in neuro-oncology. Recent research emphasizes the role of the tumor microenvironment (TME) and immune modulation in glioma progression and resistance. Despite these advances, a comprehensive bibliometric analysis of research trends in glioma chemoradiotherapy resistance over the past two decades is lacking. This study aims to systematically evaluate the research landscape, identify emerging hotspots, and provide guidance for future investigations.

**Methods:**

Articles on glioma chemoradiotherapy resistance published between 2003 and 2023 were retrieved from the Web of Science Core Collection, resulting in 4,528 publications. Bibliometric tools, including VOSviewer, CiteSpace, and R packages such as bibliometrix and ggplot2, were used to analyze co-authorship networks, keyword evolution, and citation bursts to identify collaboration patterns, thematic developments, and influential contributions.

**Results:**

Publication output increased significantly between 2013 and 2022, peaking at 650 articles in 2022. Over 1,000 institutions from 88 countries contributed to this research. The United States, Switzerland, and Germany showed the highest citation impact, while China led in publication volume but demonstrated relatively lower citation influence. The research focus has shifted from traditional topics such as the “MGMT gene” to emerging areas including the “tumor microenvironment,” “immune infiltration,” and “nanoparticles.” The androgen receptor was identified as a promising but underexplored therapeutic target.

**Conclusions:**

Research on glioma chemoradiotherapy resistance has seen substantial growth, with increasing emphasis on immune modulation, the tumor microenvironment, and novel therapeutic targets such as the androgen receptor. This study represents the first comprehensive bibliometric analysis of this field, providing a detailed overview of research trends and potential directions for future studies. The findings highlight the need for strengthened international collaboration and multidisciplinary approaches to address the challenges of therapeutic resistance in glioma.

## Introduction

Gliomas are the most prevalent and aggressive malignant tumors of the central nervous system (CNS), accounting for over 70% of all CNS tumors (1). Despite significant advancements in treatment, the prognosis for glioma patients remains poor (2). The standard approach involves maximal surgical resection, followed by radiotherapy and chemotherapy, with temozolomide (TMZ) being the most commonly used agent (3). Chemoradiotherapy induces DNA damage leading to apoptosis in tumor cells (4, 5). However, high recurrence rates and resistance to therapies continue to pose significant challenges (6).

TMZ plays a crucial role in treatment, increasing median survival from 12.1 to 14.6 months and raising two-year survival rates from 10.4% to 26.5% (7). Nevertheless, resistance to TMZ, primarily mediated by O6-methylguanine-DNA-methyltransferase (MGMT) repair mechanisms, remains a significant obstacle (8). Additional DNA repair pathways, such as base excision repair (BER) and mismatch repair (MMR), also contribute to therapeutic resistance (9–11). Furthermore, autophagy, apoptotic signaling pathways, and the tumor microenvironment (TME) are increasingly recognized as critical factors in TMZ resistance (8, 12).

The TME including tumor-associated macrophages (TAMs), microglia, neutrophils, myeloid-derived suppressor cells (MDSCs), and T cells interacts with glioma cells to promote tumor growth and therapeutic resistance (13). Current research is exploring MGMT inhibitors, DNA repair pathway inhibitors, and combination therapies to overcome resistance (14, 15).

Despite these advancements, comprehensive bibliometric analyses focusing on glioma chemoradiotherapy resistance are limited. Bibliometric studies provide quantitative and qualitative assessments of scientific literature, offering valuable insights into research trends and collaborations (16, 17). In this study, we use bibliometric tools to systematically analyze the research landscape, pinpoint key focus areas, and outline potential future directions in glioma chemoradiotherapy resistance.

## Materials and methods

### Data sources and search strategy

Bibliographic data were obtained from the Science Citation Index Expanded (SCIE) within the Web of Science Core Collection (WoSCC) (18). A systematic search strategy was developed to ensure a comprehensive literature review of glioma chemoradiotherapy resistance and its underlying mechanisms.

Search Query:

Keywords for Disease: (“Glioma” OR “Glioblastoma Multiforme” OR “GBM” OR “Glioblastoma”).

Keywords for Resistance: (“Chemoradiotherapy Resistance” OR “Radioresistance” OR “Chemoresistance” OR “Temozolomide Resistance” OR “Adaptive resistance” OR “Acquired resistance” OR “TMZ” OR “Temozolomide” OR “TMZ resistance”).

Keywords for Mechanisms: (“MGMT Promoter Methylation” OR “DNA Repair Mechanisms” OR “Cancer Stem Cells” OR “Tumor Microenvironment” OR “Epigenetic Alterations” OR “Signal Transduction Pathways” OR “PI3K/Akt/mTOR Pathway” OR “RAS/RAF/MEK/ERK Pathway” OR “Apoptosis and Autophagy” OR “Cell Cycle Dysregulation” OR “Immunotherapy” OR “Targeted Therapy” OR “Biomarkers of Resistance” OR “Molecular Profiling” OR “Precision Medicine” OR “Radiosensitizers” OR “MicroRNA” OR “Long Non-coding RNA” OR “Blood-Brain Barrier” OR “Tumor-associated macrophages” OR “Post-translational modification” OR “Methylation” OR “Acetylation” OR “Ubiquitination” OR “Clinical trial” OR “Cell cycle capture” OR “chemosensitivity”).

**Figure 1:**
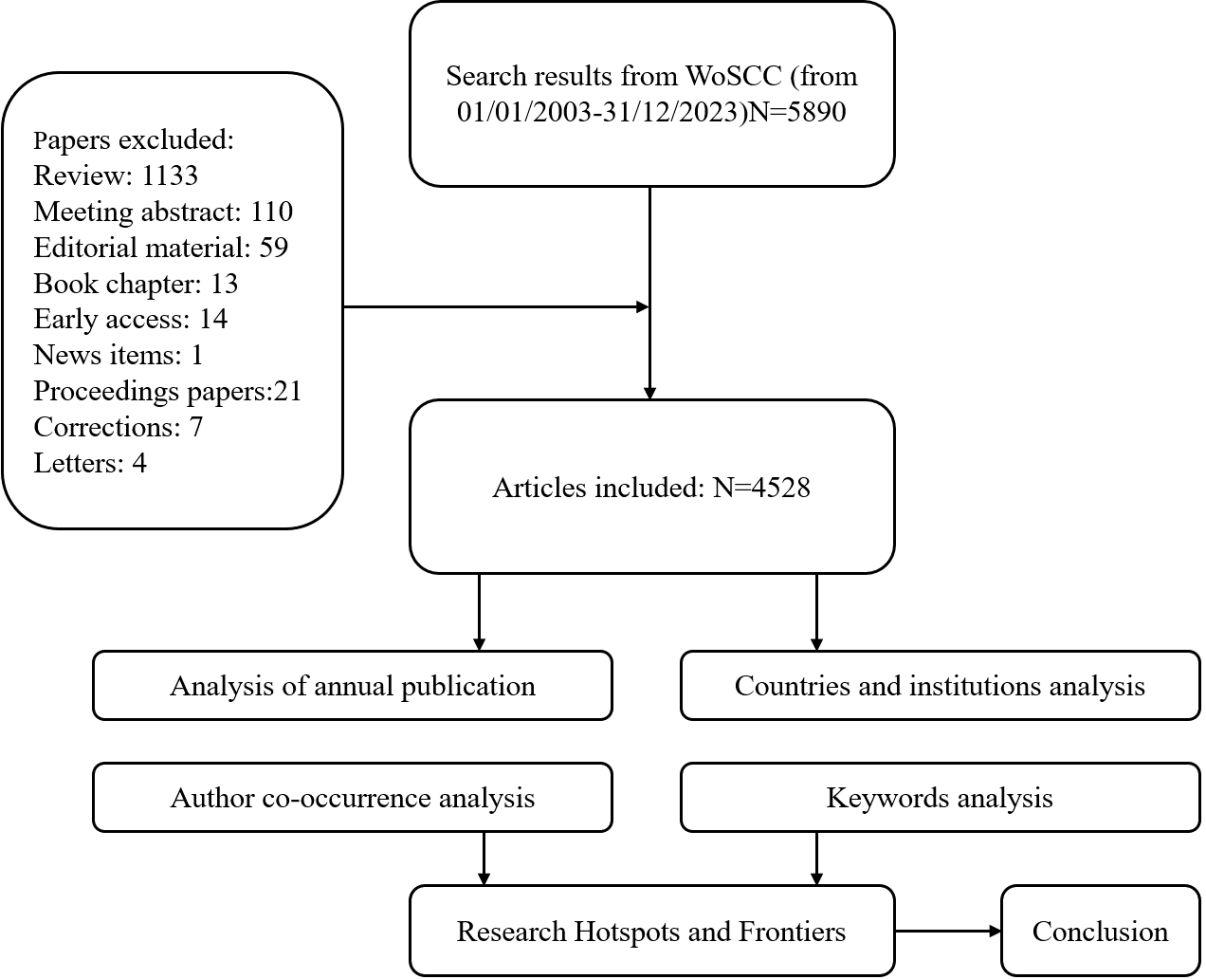
Strategy for data search and analysis.

The search was restricted to articles and reviews published in English between 2003 and 2023. A total of 5,890 documents were retrieved. After excluding conference abstracts, book chapters, and non-article documents, 4,528 articles remained for bibliometric analysis and visualization. The flow of the search and exclusion process is illustrated in **Figure 1**. The search was finalized on April 2, 2024.

### Data extraction and analysis

This study analyzed the bibliometric characteristics of publications related to chemoradiotherapy resistance in glioma, focusing on publication year, geographical distribution, institutional contributions, journals, core authors, keywords, and key references. Bibliometric analyses and network visualizations were conducted using VOSviewer (version 1.6.20, Leiden University), CiteSpace (version 6.2.6, Drexel University), and the bibliometrix package in R (version 4.2.0, R Foundation). Temporal trends in publication volume were analyzed by fitting curves using the model ƒ(*x*)=  k/[1 + a * e^(− b * x)] to predict future literature accumulation (19). Scimago Graphica was used to map the distribution and connections of countries/regions.

Co-authorship and co-occurrence analyses were conducted using VOSviewer, CiteSpace, and Microsoft Excel 2019. The Bibliometrix package was utilized to create thematic maps and analyze the evolution of keyword themes. A thesaurus file in VOSviewer was employed to merge variant terms and standardize capitalization. In the visualizations, nodes represented entities such as countries and regions, institutions, or researchers, while links between them indicated relationships evaluated by total link strength. Specific thresholds for items included in the VOSviewer maps are provided in the Results section. CiteSpace parameters were set as follows: time slicing of one year, selection criteria of the top 50 cited or co-occurring items per slice (g-index: k = 15), and pruning using pathfinder and merged network pruning methods.

## Results

### Trends in publication and citation

**Figure 2:**
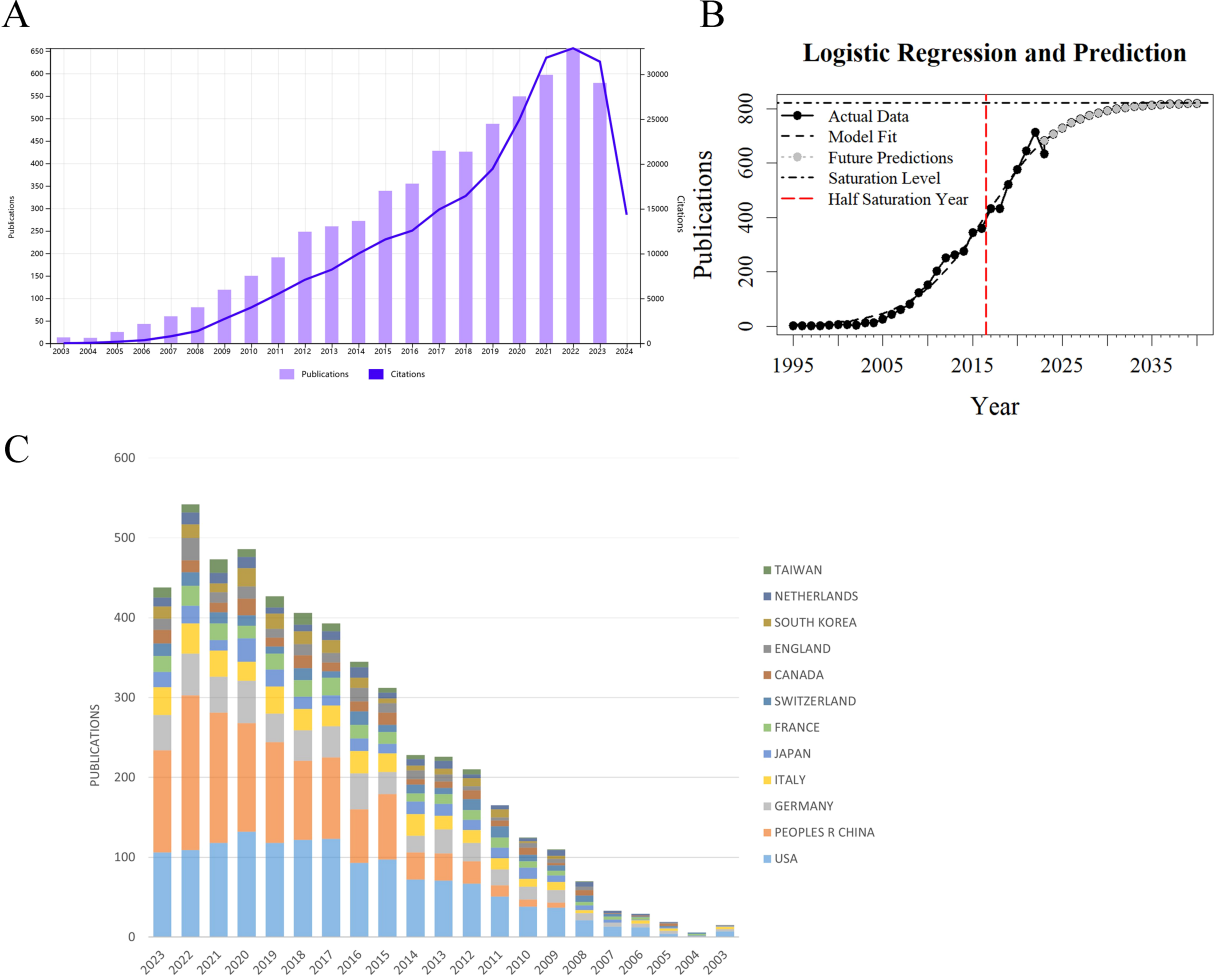
Trends in publication volume and citation count for research on glioma chemoradiotherapy resistance from 2003 to 2023 **(A)** Annual publication and citation trends for glioma chemoradiotherapy resistance: The purple bar graph represents the annual publication volume, while the blue line graph represents the annual citation count. **(B)** Model-fitted curves of the cumulative number of publications **(C)** Annual publication volume by country: This graph shows the annual publication volume distribution across different countries or regions in the field.

Based on data from the Web of Science (WoS) Core Collection, 4,528 articles on glioma chemoradiotherapy resistance were indexed between 2003 and 2023, accumulating a total of 205,658 citations. The average citations per article were 45.42, and the H-index was 167. The top 100 most-cited papers accounted for 33.68% of total citations, averaging 692.58 citations each. The top 50 papers contributed 27.05% of citations, averaging 1,112.46 citations per paper. **Figure 2A** shows the annual publication trends. From 2003 to 2007, publication and citation grew slowly. Since 2013, publications increased significantly, peaking at nearly 650 articles in 2022. Citation counts rose sharply after 2018, exceeding 30,000 in 2022. The decline in publications and citations in 2023 may be due to incomplete data collection. Although the WoS has indexed these publications since 1995, our analysis focuses on 2003 to 2023 for a comprehensive overview of recent trends. The logistic growth curve f(x) = 821.62/[1 + 187.42 * exp(-0.243 * (x - 1995))] ​ models the global publication accumulation (**Figure 2B**), suggesting that the field will sustain a favorable development trend over an extended period, the growth trend is beginning to level off. The USA and China have maintained high levels of academic output over the past decade, with China showing a particularly notable increase in research productivity, surpassing other countries in publication volume (**Figure 2C**).

### Distribution of publication and citation metrics

**Figure 3:**
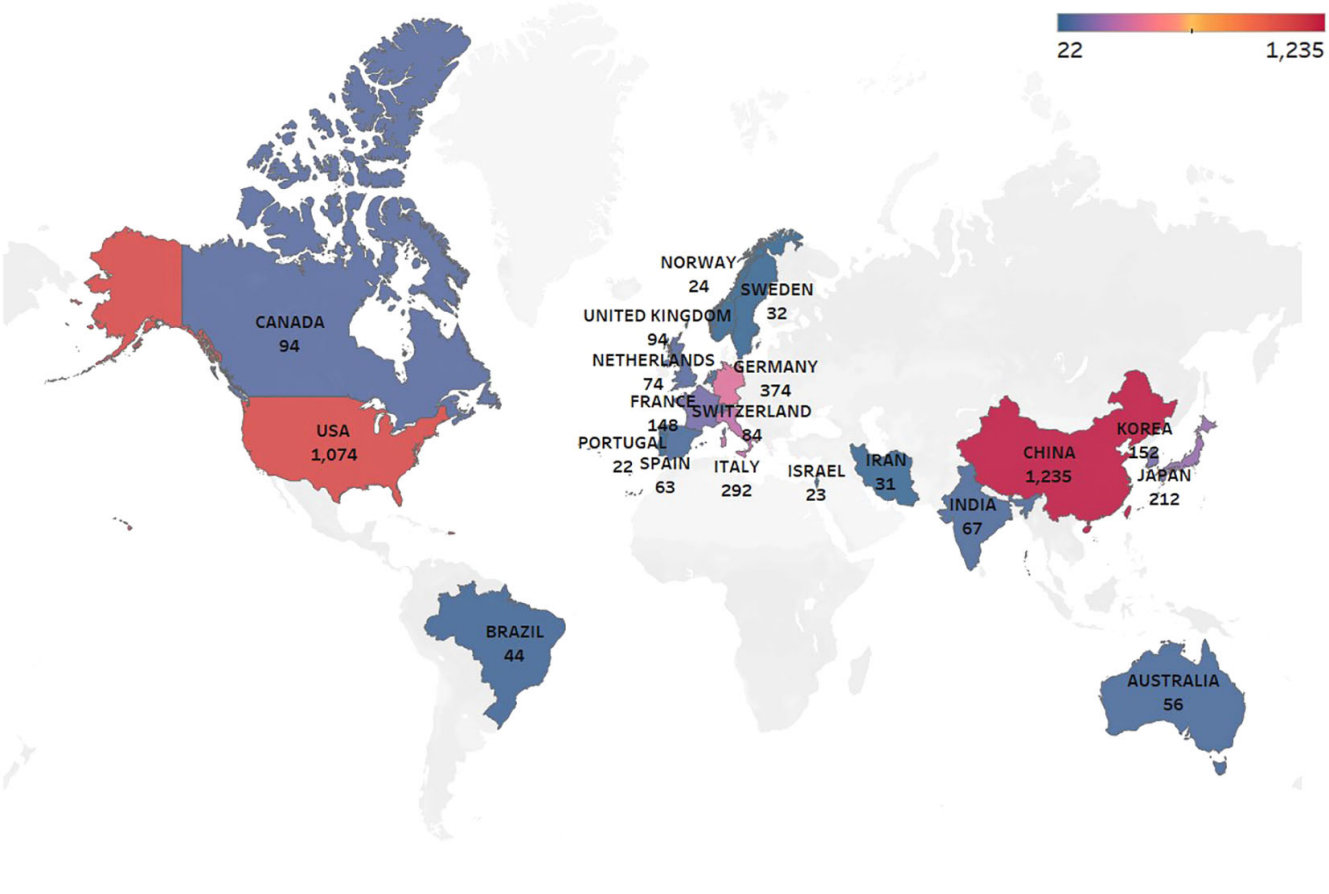
Geographic distribution of publications by country: The color intensity indicates the publication volume, with darker shades representing higher volumes.

Key bibliometric indicators such as the H-index, which reflects both productivity and impact, and citation bursts, which highlight emerging research trends, are used to analyze the research landscape. Research on glioma chemoradiotherapy resistance has been conducted in 88 countries and regions. The contributions from international collaborations are analyzed separately, and their spatial distribution is visualized in a heat map (**Figure 3**). **Table 1** presents the top 15 countries with the highest publication counts. China leads with 1,235 publications (27.3%), followed by the United States with 1,074 publications (23.7%) and Germany with 374 publications (8.3%). Despite having the highest number of publications, China has relatively lower total citations, average citations per paper and H-index values compared to the United States and Switzerland. The United States ranks highest in critical metrics such as total citations, average citations per paper, and H-index, demonstrating its leading influence in the field. Switzerland also ranks prominently in citation metrics, boasting an H-index of 84. The top 10 most-cited articles account for a total of 32,823 citations, representing 15.56% of the total citations in the field. The most-cited article, authored by Stupp and Hegi (20), has garnered 5,799 citations, averaging 362.44 citations per year. The most-cited recent article, published by (21), has accumulated 966 citations (**Table 2**).

**Table 1:** Top 15 most productive countries and regions.

Country	N	%	Total citations	Average citations	H-index
China	1235	27.3	30585	24.8	24
USA	1074	23.7	74153	69	69
Germany	374	8.3	17156	45.9	45
Italy	292	6.4	11090	38	37
Japan	212	4.7	6029	28.4	28
South Korea	152	3.4	4012	26.4	26
France	148	3.3	5584	37.7	37
Canada	94	2.1	5115	54.4	54
England	94	2.1	3487	37.1	37
Switzerland	84	1.9	22215	264.5	84
Netherlands	74	1.6	4711	63.7	63
India	67	1.5	1961	29.3	29
Spain	63	1.4	2277	36.1	36
Australia	56	1.2	1751	31.3	31
Brazil	44	1	977	22.2	22

**Table 2:** Top 10 most highly cited publications.

Title	DOI	Source	Publication date	Total citations
Effects of radiotherapy with concomitant and adjuvant temozolomide versus radiotherapy alone on survival in glioblastoma in a randomized phase III study: 5-year analysis of the EORTC-NCIC trial (20)	10.1016/S1470-2045(09)70025-7	Lancet Oncol	May 2009	5799
Comprehensive genomic characterization defines human glioblastoma genes and core pathways (22)	10.1038/nature07385	Nature	Oct 2008	5797
MGMT gene silencing and benefit from temozolomide in glioblastoma (23)	10.1056/NEJMoa043331	N Engl J Med	Mar 2005	5299
Glioma stem cells promote radioresistance by preferential activation of the DNA damage response (24)	10.1038/nature05236	Nature	Dec 2006	4854
An integrated genomic analysis of human glioblastoma Multiforme (25)	10.1126/science.1164382	Science	Sep 2008	4471
A restricted cell population propagates glioblastoma growth after chemotherapy (26)	10.1038/nature11287	Nature	Aug 2012	1655
Effect of Tumor-Treating Fields Plus Maintenance Temozolomide vs Maintenance Temozolomide Alone on Survival in Patients With Glioblastoma A Randomized Clinical Trial (27)	10.1001/jama.2017.18718	JAMA	Dec 2017	1438
Analysis of gene expression and chemoresistance of CDI33+ cancer stem cells in glioblastoma (28)	10.1186/1476-4598-5-67	Mol Cancer	Dec 2006	1417
A single dose of peripherally infused EGFRvIII-directed CAR T cells mediates antigen loss and induces adaptive resistance in patients with recurrent glioblastoma (29)	10.1126/scitranslmed.aaa0984	Sci Transl Med	Jul 2017	1127
Management of glioblastoma: State of the art and future directions (21)	10.3322/caac.21613	CA Cancer J Clin	Jul 2020	966

### Network analysis of country/region and institutional collaboration

**Figure 4:**
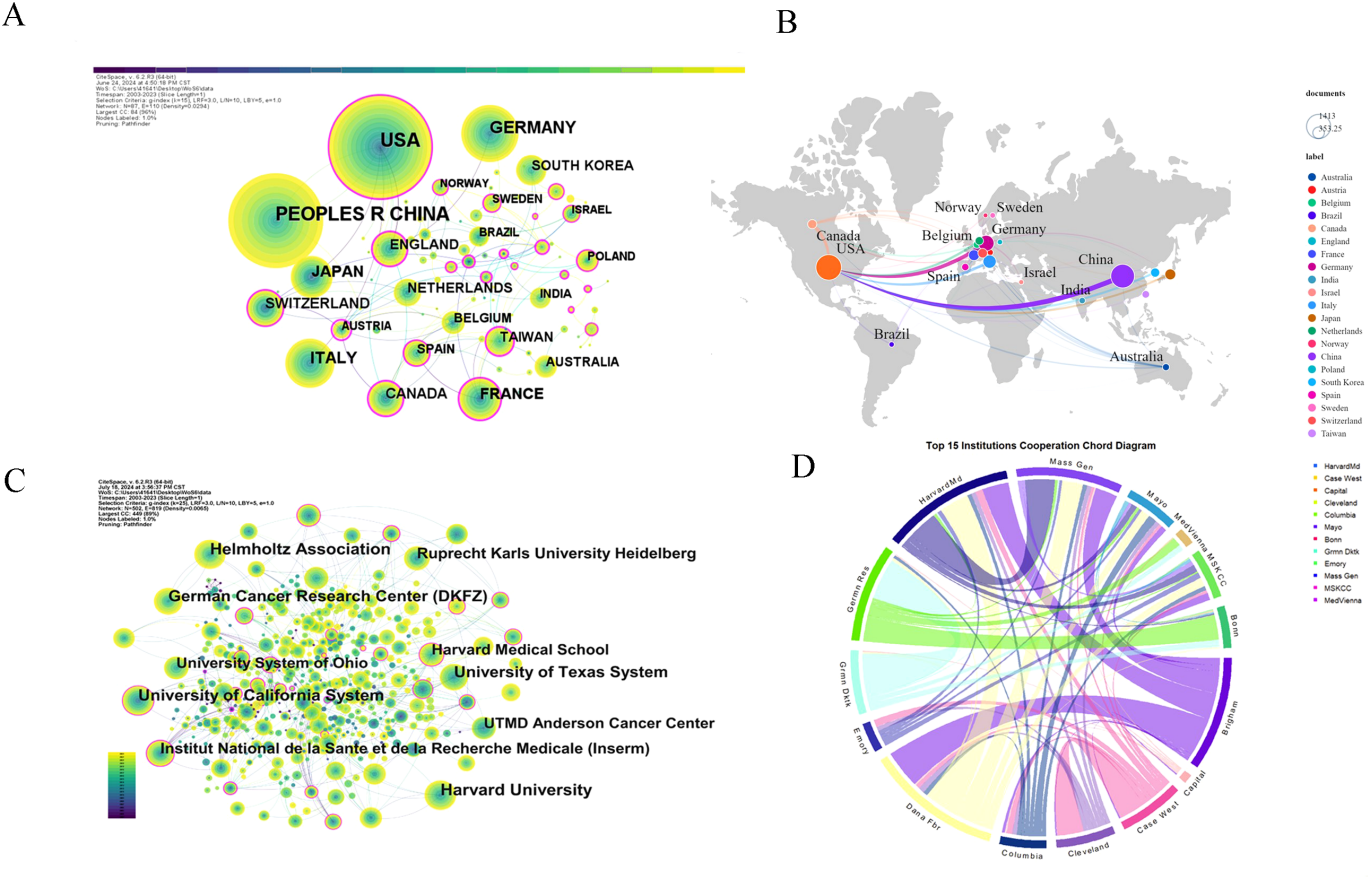
Co-occurrence relationships between countries and institutions. **(A)** Country cooperation network: Illustrates collaborative networks among countries researching glioma chemoradiotherapy resistance. **(B)** Country collaboration relationships: Displays specific collaborative relationships between countries within this research field. **(C)** Institutional collaboration network: Highlights collaborations between major research institutions focusing on glioma chemoradiotherapy resistance. **(D)** Top 15 collaborative institutions: Lists the leading institutions with significant cooperative interactions in this research area.

**Figure 4** illustrates the global collaboration network, with node size representing publication volume and line thickness indicating collaboration strength. The network comprises 87 nodes and 110 links, with a density of 0.0294 (**Figure 4A**). The United States plays a central role in collaborations, particularly with Germany, France, and Switzerland, and maintains strong ties with other European countries, including Italy and the UK (**Figure 4B**).

A total of 4,528 articles were published by 4,830 institutions and **Table 3** lists the top 15 most productive institutions. Harvard University leads with 278 publications (6.14%), followed by the Helmholtz Association (Germany) with 232 publications (5.12%) and the University of California System with 225 publications (4.97%). Among the top 15 institutions, eight are from the United States, three from Germany, and two each from France and Switzerland, highlighting the geographical concentration of leading research hubs in these countries.

**Table 3:** Top 15 Institutions by number of publications.

Affiliations	Record Count	% of 4528
Harvard University	278	6.14
Helmholtz Association	232	5.12
University Of California System	225	4.97
University Of Texas System	207	4.57
German Cancer Research Center Dkfz	198	4.37
Institut National De La Sante Et De La Recherche Medicale Inserm	186	4.11
Harvard Medical School	163	3.60
Ruprecht Karls University Heidelberg	161	3.56
Utmd Anderson Cancer Center	153	3.38
University Of Zurich	148	3.27
University System Of Ohio	144	3.18
Mayo Clinic	141	3.11
University Zurich Hospital	138	3.05
Dana Farber Cancer Institute	133	2.94
Centre National De La Recherche Scientifique Cnrs	129	2.85

**Figures 4C, D** depict the institutional collaboration network in glioma research, emphasizing key institutions and their relationships. Strong partnerships between the United States and Germany highlight robust transatlantic collaboration. The collaboration chord diagram (**Figure 4D**) identifies central nodes such as Harvard Medical School, Massachusetts General Hospital, Mayo Clinic, the German Cancer Research Center, Columbia University, MD Anderson Cancer Center, and Seoul National University, all with high connectivity.

### Analysis of high-contribution journals, leading researchers, and co-cited journals

**Figure 5:**
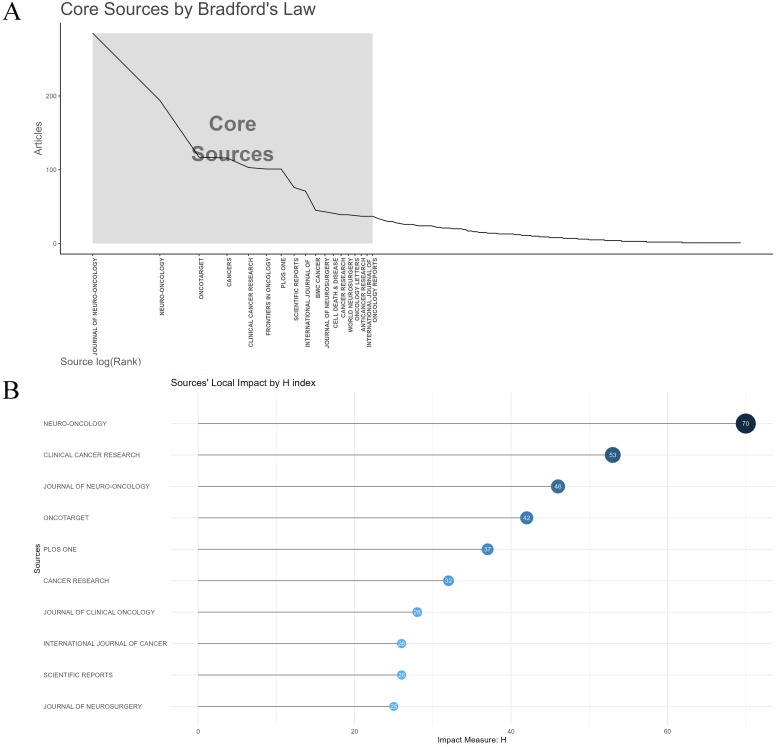
Analysis of core journals and impact metrics in glioma chemoradiotherapy resistance research. **(A)** Core sources by Bradford’s law; **(B)** Sources’ Local Impact by H-index.

The top 15 journals published 1,300 papers on glioma chemoradiotherapy resistance, representing 28.71% of total publications (**Table 4**). Journal of Neuro-Oncology leads in publication count (285 papers), while Neuro-Oncology has the most citations (13,144) and the highest H-index (70). According to Bradford’s Law, core journals like Journal of Neuro-Oncology and Oncotarget play a significant role in this field (**Figure 5A**), while their local impact within glioma research is further evaluated in **Figure 5B**. Neuro-Oncology appears to be the most influential journal in glioma chemoradiotherapy resistance research.

**Table 4:** The top 15 related popular journals.

Journal	N	%	Total citations	Average citations	H-index	IF-2024	JCR
Journal Of Neuro-Oncology	285	6.29	7745	27.18	46	3.2	Q2
Neuro-Oncology	194	4.28	13144	67.75	70	16.4	Q1
Oncotarget	117	2.58	4862	41.56	42	2.5	Q2
Cancers	116	2.56	1488	12.83	22	6.6	Q1
Clinical Cancer Research	103	2.27	8623	83.72	53	13.8	Q1
Plos One	101	2.23	4237	41.95	37	3.7	Q2
Scientific Reports	76	1.68	2232	29.37	26	4.6	Q1
Bmc Cancer	45	0.99	1228	27.29	22	3.9	Q2
Journal Of Neurosurgery	43	0.95	1695	39.42	25	5.1	Q1
Cell Death & Disease	41	0.91	1579	38.51	22	9.2	Q1
Cancer Research	39	0.86	4303	110.33	32	13.3	Q1
International Journal Of Cancer	37	0.82	2623	70.89	26	7.4	Q1
Oncology Reports	37	0.82	1260	34.05	22	3.8	Q2
International Journal Of Radiation Oncology Biology Physics	34	0.75	2132	62.71	22	7	Q1
Journal Of Clinical Oncology	32	0.71	9081	283.78	28	45.3	Q1

In terms of leading researchers, the top 10 authors were ranked based on publication count, citations, and H-index (**Table 5**). Michael Weller from the University of Zurich leads with 92 publications, 26,620 citations, and an H-index of 58, followed by Wolfgang Wick and Roger Stupp. These authors represent Switzerland, the USA, and Germany, contributing 11.64% of total publications but 66.49% of total citations, demonstrating their substantial impact on the field.

**Table 5:** Top 10 core authors by number of publications.

Rank	Authors	Organizations	Country	Publications	Citations	H- index
1	Weller M	University Hospital and University of Zurich	Switzerland	92	26620	58
2	Wick W	Department of Neurooncology, University of Heidelberg	Germany	75	11717	44
3	Stupp R	Department of Neurological Surgery, Northwestern University Feinberg School of Medicine	USA	55	23556	40
4	Reifenberger G	Institute of Neuropathology, University Hospital Düsseldorf and Medical Faculty	Germany	51	6640	32
5	Brandes AA	Department of Medical Oncology, University Hospital, Padua	Italy	44	13390	31
6	Von Deimling A	Department of Neuropathology, Ruprecht-Karls-University	Germany	46	5080	30
7	Gorlia T	European Organization for Research and Treatment of Cancer	Belgium	44	16742	29
8	Hegi ME	Department of Clinical Neurosciences, University Hospital Lausanne	Switzerland	35	19918	28
9	Sarkaria JN	Department of Radiation Oncology, Mayo Clinic	USA	46	2397	28
10	Van Den Bent MJ	Department of Neurology, Brain Tumor Center, Erasmus MC Cancer Institute	Netherlands	39	10691	26

**Figure 6:**
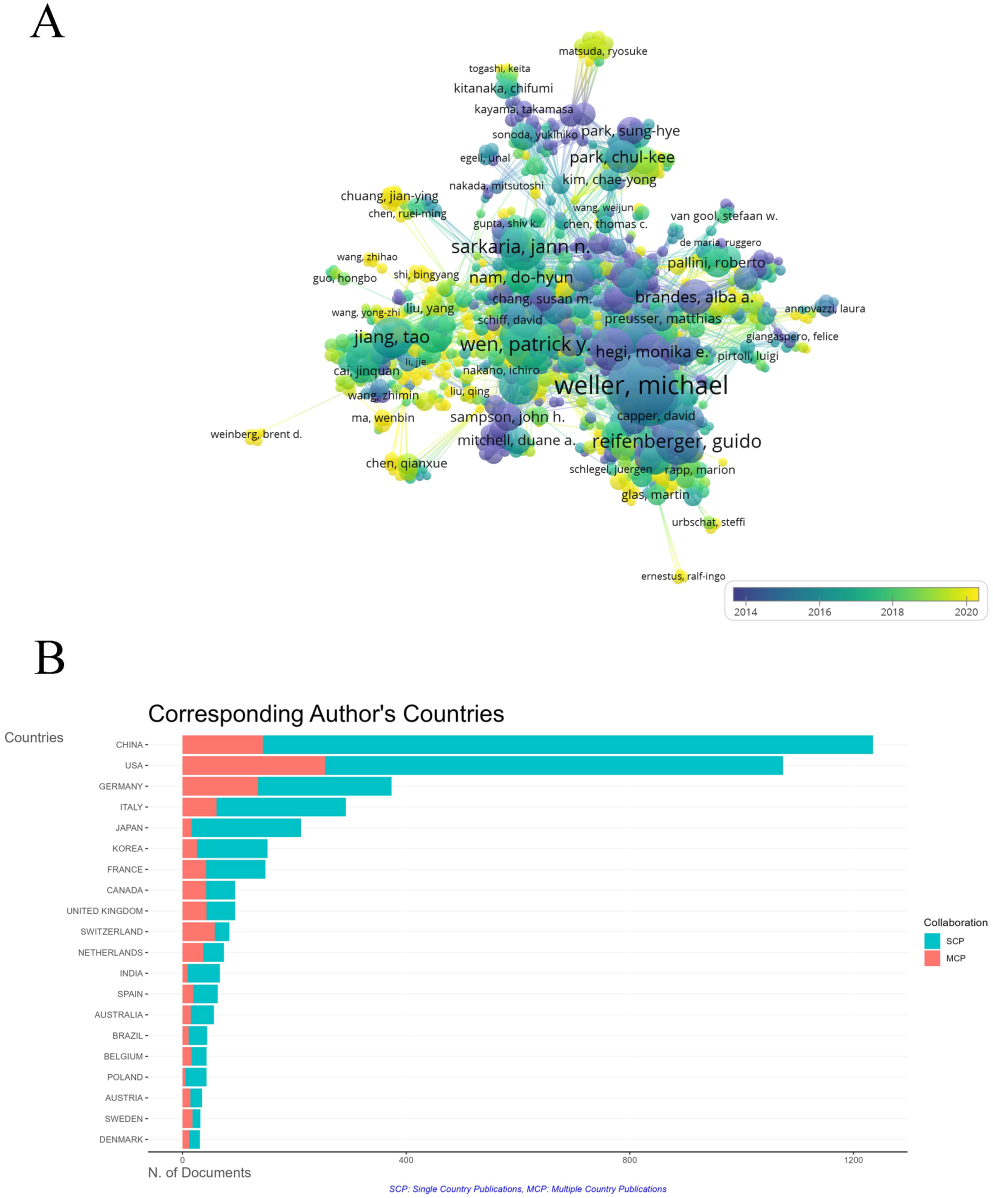
The co-occurrence of authors. **(A)** Overlay visualization of co-authorship relationships between authors. The analysis method was Linlog/modularity. The weight was citations. Scores are the average year of publication. The thickness of the lines indicates the strength of the relationships. The colors of the circles represent the average year of publication. **(B)** Co-responding author’s countries based on their publication volume in the field. The green bars represent Single Country Publications (SCP), The red bars represent Multiple Country Publications (MCP).

A co-authorship map generated by VOSviewer shows collaboration networks of 27,310 authors, with Michael Weller, Guido Reifenberger, and Roger Stupp as key figures in glioma research (**Figure 6A**). Chinese researchers, including Jiang Tao, have become more active since 2016, though China’s rate of multinational collaboration remains relatively low (**Figure 6B**).

**Figure 7:**
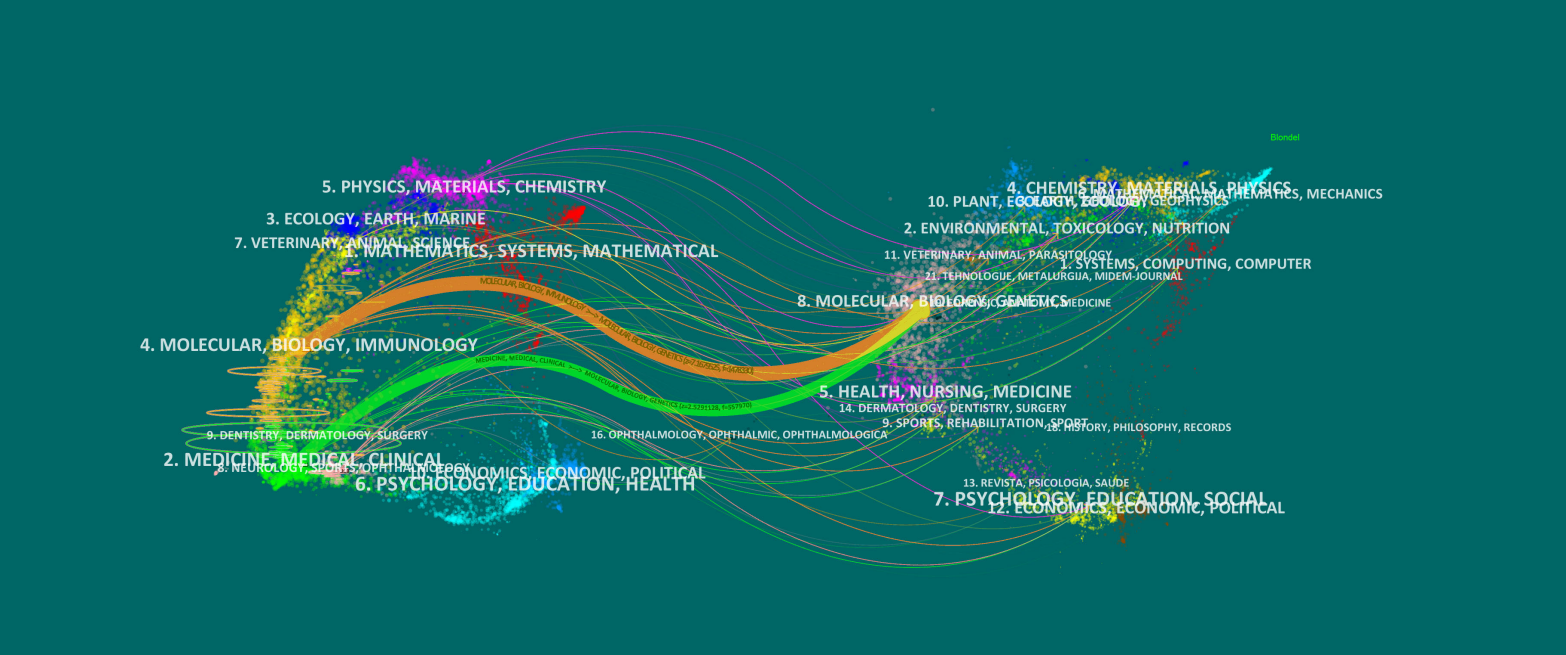
Dual-map overlay of the journals on research (2003–2023). Each point on the map represents a journal. The left side of the map shows the citing journals, and the right part presents the cited journals. Colored paths represent reference relationships, with thicker lines represent the main paths.

**Figure 7** presents the dual-map overlay, illustrating the citation relationships in glioma-related research. Journals on the left represent the citing map, while those on the right show the cited map, with curved lines indicating citation flows. Publications in Molecular Biology and Immunology are mainly influenced by journals in Molecular Biology and Genetics (z = 7.17, f = 1,478,330), following the orange trajectory. Similarly, articles in Medicine, Medical, and Clinical fields are influenced by journals in Molecular Biology and Genetics (z = 2.53, f = 557,970), shown by the green trajectory. This highlights the strong influence of molecular biology and genetics in glioma research, reflecting its interdisciplinary nature and its integration into clinical studies.

### Keywords and co-citation analysis

**Figure 8:**
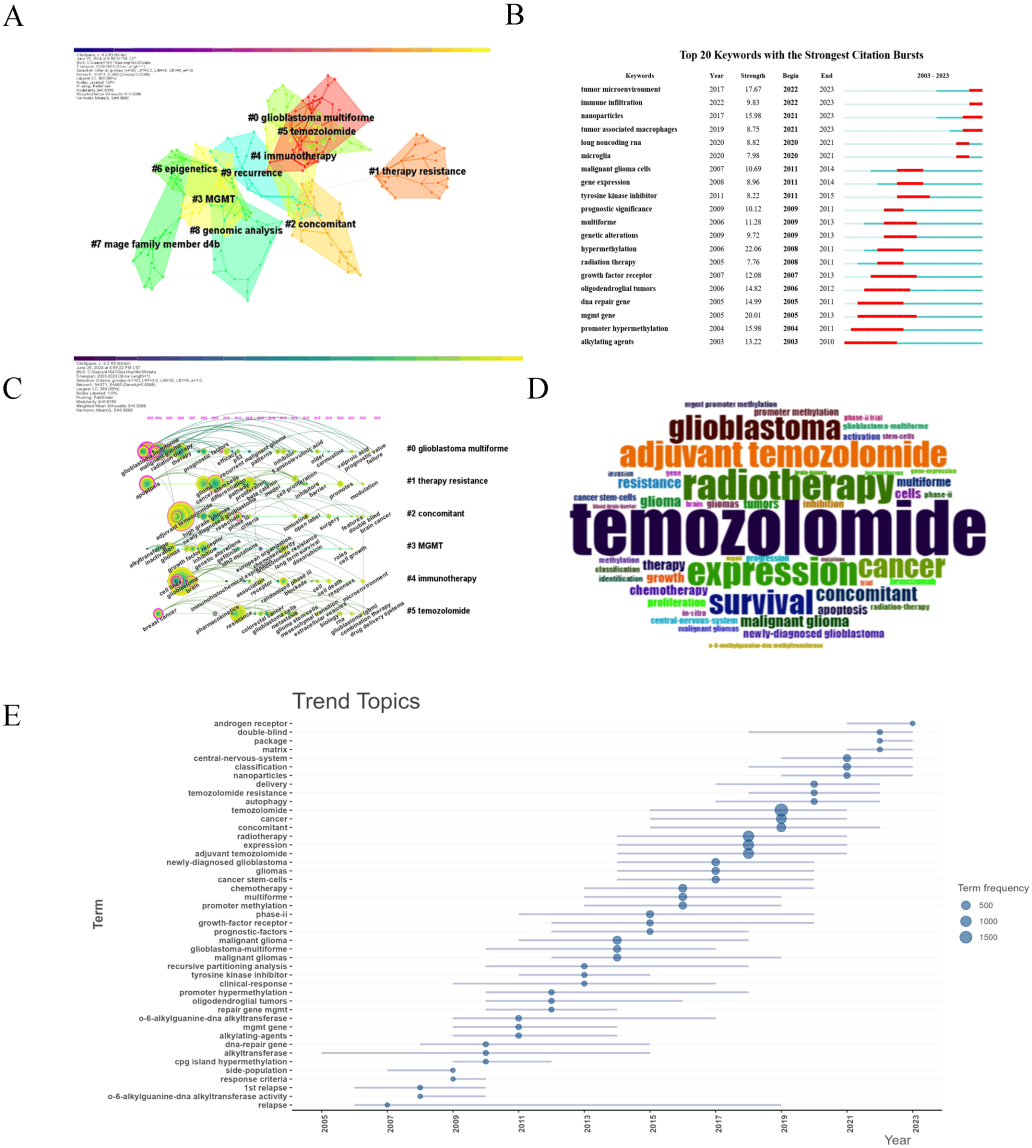
Keyword co-occurrence analysis. **(A)** The top 10 clusters of keywords. Each color region represents a cluster, and each node denotes a keyword. Areas with the same color represent a cluster with the same topic. Silhouette S = 0.9288. Modularity Q = 0.8156. **(B)** Top 20 keywords with the strongest citation bursts from 2003 to 2023. The “Strength” represents the strength of citation bursts. The red segment represents the begin and end year of the burst duration. **(C)** Timeline view of the keywords. Each circle represents a keyword, and circles on the same line represent a cluster with the same topic. The position of each circle represents the time it first appeared. The size of the circle is proportional to the frequency of keyword occurrences. **(D)** Word cloud generated by R, word size representing frequency. **(E)** Trend topics from 2003~2023, blue dots of different sizes represent word frequency.

We performed keyword co-occurrence and co-citation analyses to uncover key research trends and foundational studies in glioma chemoradiotherapy resistance. The keyword co-occurrence network (507 nodes, 1213 links, Q = 0.7558, S = 0.8868) highlighted prominent terms such as “temozolomide,” “radiotherapy,” “resistance,” and “apoptosis” (**Figure 8A**, **Table 5**). Ten major clusters were identified, with the largest focusing on topics like #0 glioblastoma, #1 treatment resistance, #3 MGMT, and #4 immunotherapy, reflecting critical challenges in glioma therapy.

Keyword frequency analysis (**Table 6**) confirmed “temozolomide” as the most frequent term, highlighting its critical role in glioma treatment. The timeline analysis (**Figures 8B, C**) showed a shift from early research on glioblastoma and chemotherapy to recent interest in temozolomide resistance, MGMT gene methylation, and immunotherapy. From 2018-2023, topics like immune infiltration, nanoparticles, and tumor-associated macrophages gained prominence, reflecting the rise of precision therapies. The word cloud (**Figure 8D**) confirmed these themes, while trend analysis (**Figure 8E**) highlighted increased focus on “cancer stem cells” and “precision medicine.”

**Table 6:** Top20 keywords in the publications on the “mechanisms of glioma drug resistance” according to frequency.

Rank	Keywords	Frequency	Centrality
1	temozolomide	1750	0.17
2	radiotherapy	966	0.04
3	expression	914	0.18
4	adjuvant temozolomide	835	0.14
5	glioblastoma	779	0.03
6	survival	778	0.07
7	concomitant	477	0.07
8	glioblastoma multiforme	436	0.22
9	malignant glioma	414	0.12
10	resistance	340	0.06
11	cancer stem cells	320	0.11
12	chemotherapy	320	0.13
13	apoptosis	300	0.36
14	multiforme	289	0.02
15	proliferation	264	0.01
16	promoter methylation	252	0
17	inhibition	248	0.03
18	mgmt promoter methylation	239	0.02
19	central nervous system	212	0.11
20	malignant gliomas	205	0.08

**Figure 9:**
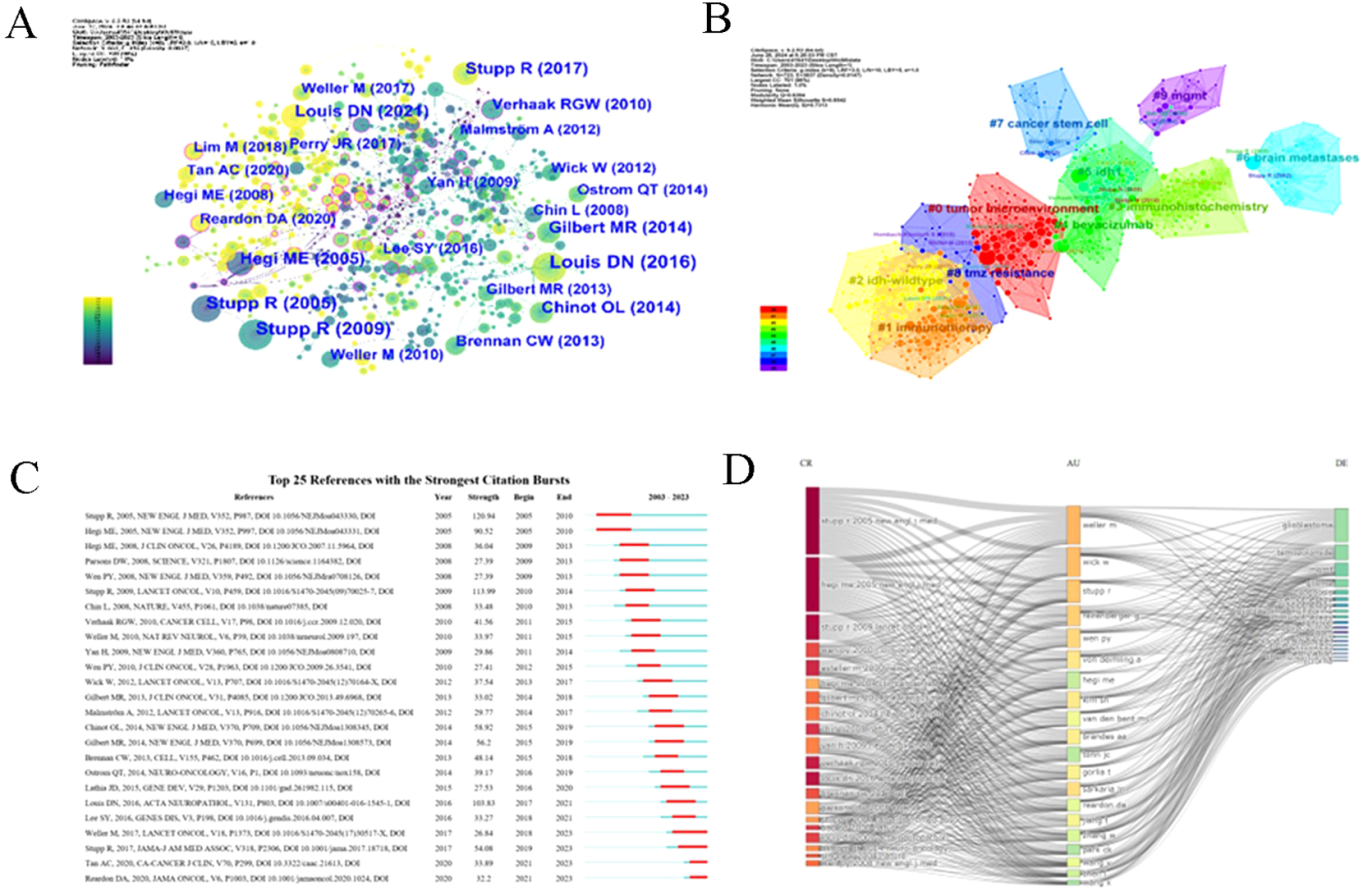
Co-cited references and burst references. **(A)** The network map of co-cited references. Nodes in the visualized network represent co-cited references. Lines between nodes represent co-cited links. **(B)** The network map of co-cited clusters. 10 clusters with diversified research themes were formed and illustrated in different colors. Areas with the same color represent a cluster with the same topic. Silhouette S = 0.8542. Modularity Q = 0.6394. **(C)** Top 25 references with the strongest citation burst from 2003-2023. The “Strength” represents the strength of citation bursts. The red segment represents the begin and end year of the burst duration. **(D)** A three-field plot illustrates the relationships between frequently cited references, key researchers, and pivotal terms.

Co-citation analysis (662 nodes, 815 links, Q = 0.6394, S = 0.8542) revealed 16 key clusters (**Figure 9A**), with the largest—#0 “tumor microenvironment” and #1 “immunotherapy”—providing insights into resistance mechanisms. Several influential studies, such as Stupp et al. (20, 30) and Louis et al. (31), showed strong citation bursts, highlighting their foundational role. Emerging fields, like immunotherapy and PD-1 inhibitors, were identified with ongoing citation bursts (**Figures 9B, C**). A three-field plot (**Figure 9D**) mapped relationships between frequently cited references, key researchers (Weller M, Wick W, Stupp R), and terms like “glioblastoma” and “MGMT,” illustrating collaborative networks driving innovation in this domain.

## Discussion

### Global research trends in glioma chemoradiotherapy resistance mechanisms

The increasing number of publications reflects heightened global attention to glioma chemoradiotherapy resistance mechanisms. Since Friedman’s seminal 1998 study on temozolomide efficacy and tumor DNA mismatch repair activity (32), research in this field has expanded significantly. Our bibliometric analysis reveals a notable rise in publications and citations, with annual publications exceeding 400 since 2017 and peaking at 650 in 2022. This surge indicates not only the maturation of theoretical frameworks but also a growing recognition of the clinical challenges posed by glioma resistance.

Countries with higher glioma incidence, primarily developed nations, have historically dominated publication output, possibly due to regional differences in tumor incidence (33). A 2012 European Journal of Cancer study highlighted global variations in malignant CNS tumor rates, with the highest in Europe, North America, and Australia/New Zealand (34). Studies in Neuro-Oncology also revealed significant incidence differences, with the highest rates in Europe and lower rates in Asia (35). This likely contributed to the United States’ increased research focus on glioma resistance mechanisms (36).

In Asia, South Korea’s 2002 nationwide CNS tumor survey highlighted differences in CNS tumor incidence compared to Western populations (37). Factors like HDI, GDP, and occupational carcinogen exposure correlate with glioma incidence, driving increased research in countries like China since 2009 (38, 39). However, global collaboration remains limited, underscoring the need for interdisciplinary research to advance glioma resistance studies and develop effective treatments.

### Hot topics in glioma chemoradiotherapy resistance mechanisms: temozolomide resistance and tumor microenvironment

Keywords highlight research hotspots, with temozolomide (TMZ) resistance and the tumor microenvironment (TME) emerging as core topics in glioma research (40, 41). TMZ, the standard chemotherapeutic for glioblastoma (GBM), often faces efficacy challenges due to resistance mechanisms (9, 19, 42–45). Despite its effectiveness, glioma cells develop resistance through various mechanisms, such as MGMT repair of TMZ-induced DNA damage, overexpression of EGFR, and mutations in Mdm2, p53, and PTEN. Strategies to overcome these include MGMT inhibitors and EGFR inhibitors, which require further clinical validation (23, 27, 46).

MGMT promoter methylation is a critical biomarker for predicting TMZ response, and developing MGMT inhibitors remains a significant research focus (24, 47). Additionally, researchers are exploring other DNA repair pathways, such as APNG, for potential therapeutic targets (48–50).

Recent research has increasingly focused on the TME, which plays a key role in glioma progression and treatment resistance (5). Immune infiltration, nanoparticle drug delivery, and tumor-associated macrophages (TAMs) are major areas of interest. TAMs in the TME contribute to tumor growth and resistance by secreting cytokines and growth factors, while the blood-brain barrier limits TMZ penetration (51). Modulating the TME to enhance immune responses is a growing field of study (52). New technologies like CRISPR-Cas9 and single-cell RNA sequencing are being used to identify and target resistant cell populations, offering new therapeutic strategies (53).

### Future research trends

Our bibliometric analysis reveals the evolving research focus in glioma chemoradiotherapy resistance. Prior to 2010, studies primarily centered on foundational mechanisms such as “promoter hypermethylation,” “DNA repair genes,” and “alkylating agents.” Between 2011 and 2015, the focus shifted toward treatment strategies, including the “MGMT gene,” “growth factor receptors,” and “tyrosine kinase inhibitors.” Since 2016, interdisciplinary research has become increasingly prominent, with key topics such as the “tumor microenvironment,” “immune infiltration,” and “nanoparticles” reflecting significant advances in immunology and nanotechnology.

The keyword timeline shows “tumor microenvironment,” “combination therapy,” and “drug delivery” as the most frequent terms in 2023, expected to remain key in future research. The androgen receptor (AR) also emerges as a potential target, with studies linking AR expression to poor prognosis and increased resistance to temozolomide (TMZ) (54–60). A study (61) highlights a dual-targeted delivery system for temozolomide using a multi-responsive nanoplatform that modulates the tumor microenvironment to overcome drug resistance in glioblastoma. This innovative approach addresses key challenges such as low delivery efficiency and chemotherapy resistance, offering a promising new avenue for improving GBM treatment outcomes.

While sex hormones like estrogen and androgen are well-studied in other cancers (62, 63), their role in GBM remains unclear. Targeting AR with antiandrogen drugs presents a promising therapeutic strategy to combat treatment resistance (57, 64). Brain-penetrant antiandrogens offer potential in biomarker-guided treatments, and ongoing studies aim to refine these therapies by exploring biological sex and the immune microenvironment (65). Emerging clinical trials highlight the potential of androgen receptor (AR)-targeted therapies in glioblastoma. Researchers have initiated a trial to evaluate the safety and tolerability of enzalutamide combined with radiotherapy (RT) and TMZ (66). The study also uses Response Assessment in Neuro-Oncology (RANO) criteria to assess clinical responses and examines the pharmacokinetics of enzalutamide with TMZ. These efforts mark a key step toward integrating AR-targeted therapies into standard glioblastoma treatment, offering new possibilities for overcoming resistance and improving outcomes.

### Limitations of our bibliometric analysis

This bibliometric analysis provides valuable insights into the research landscape of glioma chemoradiotherapy resistance, but some limitations must be acknowledged. First, the data were sourced exclusively from the Web of Science database, potentially introducing bias by excluding publications indexed in other databases like Scopus or PubMed. Additionally, the search strategy excluded non-English articles, possibly leading to language bias and omission of relevant studies. Another limitation lies in the reliance on metadata and citation data rather than full-text content, meaning the analysis cannot capture detailed discussions, such as authors’ interpretations or insights into future directions. As a result, certain nuances of the field may be overlooked. Finally, citation-based metrics like the H-index and citation bursts reflect research impact but not necessarily the quality or clinical relevance of studies, which may affect result interpretation. Acknowledging these limitations provides context for our findings and highlights the need for complementary approaches, such as systematic reviews or meta-analyses, to achieve a more comprehensive understanding of glioma chemoradiotherapy resistance.

## Conclusion

This study represents the first bibliometric analysis of glioma chemoradiotherapy resistance using visualization software, illustrating the current research landscape over the past 21 years. The number of published papers has shown a significant upward trend, particularly in the past decade, indicating substantial global interest in the field of glioma chemoradiotherapy resistance. Currently, the main research hotspots focus on temozolomide resistance, tumor microenvironment, and nanoparticle drug delivery systems, aiming to explore resistance mechanisms and novel therapeutic approaches. Overall, this bibliometric analysis provides valuable references for researchers, helping them to comprehensively understand the key contributors to glioma chemoradiotherapy resistance mechanisms and discover further research ideas and inspiration from the identified hotspots and frontier studies.

## Data Availability

The original contributions presented in the study are included in the article/supplementary material. Further inquiries can be directed to the corresponding authors.
